# Healthcare practitioners and students’ PEP knowledge, attitude and adherence in Southern Africa

**DOI:** 10.4102/hsag.v27i0.2036

**Published:** 2022-11-09

**Authors:** Lufuno Makhado, Ofhani P. Musekwa, Thendo G. Makhado, Reamogetse Otsheleng

**Affiliations:** 1 Department of Public Health, Faculty of Health Sciences, University of Venda, Thohoyandou, South Africa; 2 Department of Psychology, Faculty of Health Sciences, University of Venda, Thohoyandou, South Africa; 3 Department of Advanced Nursing Sciences, Faculty of Health Sciences, University of Venda, Thohoyandou, South Africa; 4 Department of Health, General Delarey Hospital, North-West Province, Lichtenburg, South Africa

**Keywords:** health care practitioners, students, Southern Africa, knowledge, attitudes, adherence

## Abstract

**Background:**

There is a high prevalence of occupational exposure among health care practitioners (HCPs) around the globe. One of the risk factors of HIV infection among HCPs is occupational exposure to human immunodeficiency virus (HIV) infection through blood or fluid from HIV-infected individual. Therefore, because of this prevalence, there is a need to have sufficient knowledge and information regarding post-exposure prophylaxis (PEP). Sufficient PEP knowledge assists individuals in utilising PEP in case of exposure to HIV.

**Aim:**

This study aims to assess the level of knowledge, attitude and adherence to PEP in HCPs in Southern Africa.

**Setting:**

The systematic review included studies conducted in Southern Africa.

**Methods:**

A systematic review was conducted. Search engines employed in this study included Google Scholar, Science Direct, PubMed, Ebscohost and JSTOR. From these, 3572 articles emerged after removing duplications, and article screening was guided by inclusion and exclusion criteria and outlined on a PRISMA flow chart. Ultimately, the study included 13 articles. A critical appraisal skills programme was applied for the quality assessment of the eligible studies.

**Results:**

Studies included in this review revealed that most participants have adequate knowledge regarding PEP (*n* = 6). However, it was revealed that poor adherence occured because most HCPs did not complete PEP.

**Conclusion:**

Health care practitioners have adequate knowledge of PEP with varying levels of adherence to PEP. Therefore, more awareness illustrating the importance of adhering to PEP is needed.

**Contribution:**

There is an adequate level of knowledge regarding PEP found in the included studies although not satisfactory in Southern Africa given the participants are HCPs, and marked inappropriate practices affect adherence.

## Introduction and background

Global statistics show the prevalence of possible occupational human immunodeficiency virus (HIV) exposure and infection within one’s (Health Care Practitioner [HCP]) career to be 56.2%, and considering sociodemographic factors, the prevalence is about 54.8% (Mengistu, Tolera & Demmu 2021). Because of the manner of work, it is common for HCPs to be exposed to blood, needle stick injuries or the possibility of infection. Annually, 35.7 million HCPs are subject to percutaneous exposures (Shil & Upashe 2020). In Africa, the prevalence of occupational exposure is approximately 92% among HCPs depending on the year (Mossburg et al. 2019). Post-exposure prophylaxis (PEP) was designed as a ‘reactive’ method to prevent HIV infection, serving the same purpose as pre-exposure prophylaxis (PrEP), which may be considered a ‘proactive/preventative’ measure. Post-exposure prophylaxis is a measure provided to minimise the risk of infection following potential exposure to bloodborne pathogens (Smoot 2021; Tekalign et al. 2022). Post-exposure prophylaxis includes counselling, risk assessments, relevant laboratory investigations and 4 weeks of antiretroviral treatment with follow-up and support. Thus, in the event of occupational exposure, HCPs who wish to take PEP should have adequate information and a counselling session that would assist them in fully benefiting from PEP and preventing HIV transmission (Adebimpe 2018).

Rossouw et al. (2014) claim that all cases of occupational exposure involve noticeable risk; hence, it is substantial for HCPs to take the PEP drug post-exposure. However, we find that even with the prevalence of infection, other factors affect the efficacy of PEP. These factors include pill burden, side effects and psychological distress that may influence completion rates (Sultan, Benn & Waters 2014). However, Beekmann and Henderson (2014) suggest that frequency, severity, duration and reversibility of side effects are essential considerations in formulating a prophylactic treatment regimen.

Given that Southern Africa has the highest impact of HIV and AIDS, for it to meet the sustainable development goal (SDG), goal number 3 good health and well-being target 3.3 indicating the ending of the epidemics of AIDS and other communicable diseases that by the year 2030 (World Health Organization 2022), there must be evidence of knowledge and adherence to universal precautionary measures and PEP. The description of the level and nature of HCPs’ attitudes towards PEP is also of paramount importance. Hence, an improved understanding of PEP’s knowledge, attitudes and adherence among HCPs in Southern Africa has the potential to inform researchers and policymakers to increase safety and decrease occupational infection rates. This systematic review aims to assess knowledge, attitudes and adherence to PEP among HCPs in Southern Africa. The specific objective was to review the available literature to determine the level of knowledge regarding PEP, attitudes toward PEP and the level of adherence to PEP.

## Systematic review question

What is the knowledge, attitudes and adherence to PEP among HCPs in Southern Africa?

## Methods and approach

The systematic review was conducted following a systematic review and meta-analysis guidelines (Moher et al. 2015). This type of review was selected based on its strength in identifying, appraising and synthesising all available research relevant to this study’s review question. The review methods included search strategy, inclusion and exclusion criteria, study selection and abstraction, critical appraisal and thematic analysis.

### Search strategy

Search engines adopted in this study included Google Scholar, Science Direct, PubMed, Ebscohost and JSTOR. Keyword search, Boolean search, text searching and reference search were the search methods applied in this study. Search words included:

- Google Scholar: *Knowledge, Attitude toward and adherence to PEP among health care practitioners in Sub-Saharan Africa,*
- Science Direct: *Knowledge, Attitude toward and adherence to Post-Exposure Prophylaxis, among health care practitioners in Sub-Saharan Africa,*
- PubMed: *((((((attitudes) AND (knowledge)) AND (adherence)) OR (post-exposure prophylaxis)) OR (PEP)) OR (post-exposure prophylaxis)) AND (health care practitioners)) OR (Healthcare workers)) OR (Healthcare providers)) and (Sub-Saharan Africa)),*
- Ebscohost: *Knowledge, Attitude toward and adherence to post-exposure prophylaxis among health care practitioners in Sub-Saharan Africa,*
- JSTOR: Knowledge, *Attitude toward, and adherence to PEP among health care practitioners in Sub-Sahara Africa.*

### Inclusion and exclusion criteria

This study included studies on knowledge, attitudes and adherence to PEP among HCPs (nurses/midwives and medical doctors) and students training in nursing or medicine. Furthermore, the studies included were between 2014 and 2021. Only studies conducted in Southern Africa/sub-Saharan Africa were included in the review. Only articles that were published in English were included. Articles not on knowledge, awareness or practices of PEP among HCPs were not considered for this review.

### Study selection

**FIGURE 1:**
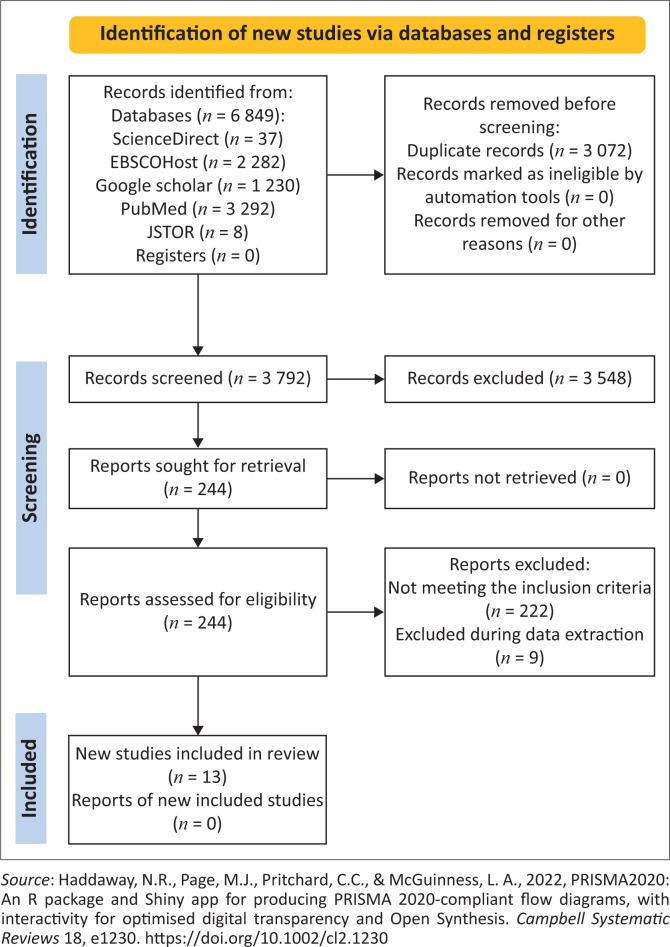
Prisma flow chart.

The researchers used the PRISMA flow chart described by Moher et al. (2009) (see Figure 1; Haddaway et al. 2020) to outline the study flow in the search results. A total number of 6849 articles emerged from the search engines. From these articles, the researchers did the topic screening, abstract screening and full-text, which resulted in duplications being removed and studies that did not meet the inclusion criteria. In the end, 13 studies were found eligible to be included in this review as they met the inclusion criteria and responded to the study’s review question, aim and focus.

### Critical appraisal of eligible studies

These included studies were critically appraised using the critical appraisal skills programme (CASP) checklist to ensure that the included studies are of good quality and conform with the research rigour of their respective approaches. Eight studies were subject to the cohort study checklist, three went through the systematic review/literature review checklist and one went through the qualitative checklist. The majority of the included studies (*n =* 11) scored above 70%, and only two studies fell below 70% in the critical appraisal (see Table 1).

**TABLE 1:** Critical appraisal skills programme study appraisal.

Authors	Study design	CASP score	%
Bareki and Tenego (2018)	Cross-sectional study	8/12	67
Aigbodion et al. (2019)	Cross-sectional descriptive	10/12	83
Ncube et al. (2014)	Cross-sectional descriptive	7/12	58
Makhado and Davhana-Maselesele (2016)	Cross-sectional descriptive	9/12	75
Rasweswe and Peu (2020a)	Descriptive study	10/12	83
McDowall and Laher (2019)	Cross-sectional survey	8/12	67
Makhado and Seekane (2020)	Systematic review	8/10	80
Kabotho and Chives (2020)	Cross-sectional study	9/12	75
Rasweswe and Peu (2020b)	Descriptive quantitative survey	10/12	83
Mossburg et al. (2019)	Systematic review	9/10	90
Mutisya (2021)	Literature Review.	8/10	80
Mabina et al. (2018)	Cross-sectional study	10/12	83
Mekgoe et al. (2019)	Phenomenological study	8/10	80

CASP, critical appraisal skills programme.

### Characteristics of the included studies

With all the studies in this review, information abstracted from the articles included the following characteristics: author and year of publication, objective/aim, country, study design, population and sample size, outcomes and limitations. Refer to Table 2 for the characteristics of the included studies.

**TABLE 2:** Characteristics of the included studies.

Author and year	Objective/aim	Country	Study design	Population and sample size	Outcomes	Limitations
Bareki and Tenego (2018)	To assess the KAP of post-exposure prophylaxis among the healthcare workers in Princess Marina Hospital, Gaborone/Botswana.	Botswana	Cross-sectional study	Registered nurses or medical doctors. A total of 247 participants.	Adequate knowledge about PEP does not guarantee uptake – poor PEP practices.	Not included.
Aigbodion et al. (2019)	To investigate the prevalence and practices pertaining to occupational blood and body fluids exposures (OBBFEs) among a select group of intern doctors.	South Africa	Cross-sectional descriptive	Intern doctors. A total of 175 participants.	OBBFE is common among intern HCW. There is adequate knowledge on PEP.	Only four hospitals were used in Gauteng; selective non-disclosure and recall bias may also apply.
Ncube et al. (2014)	To assess and evaluate knowledge and attitudes towards NO-PEP among medical students.	South Africa	Cross-sectional descriptive	First and second-year medical students. A total of 197 participants.	Knowledge among students is good, but there are concerns about those who lack understanding. There are stigma and barriers towards NO-PEP, and there is a need to raise awareness.	Not included.
Makhado and Davhana-Maselesele (2016)	To determine knowledge, insight, and uptake of occupational post-exposure prophylaxis (OPEP) among nurses caring for PLWH.	South Africa	Cross-sectional descriptive	Nurses caring for PLWH. A total of 233 participants.	The majority of nurses knew what PEP was, but several nurses did not know what it was, where to get it and more than half did not receive it when needed.	One region hospital was used; findings cannot be generalised.
Rasweswe and Peu (2020a)	To determine the occupational exposures and use of HIV (PEP).	South Africa	Descriptive study	Nurses in Gauteng. Ninety-four participants.	There is a gap in occupational exposure reporting and attaining PEP.	The study was conducted in one hospital in Gauteng. Non-despondence bias as some participants did not answer some questions.
McDowall and Laher (2019)	To investigate the cumulative incidence, knowledge, attitudes and practices pertaining to NSIs.	South Africa	Cross-sectional survey	Prehospital emergency medical service workers. A total of 240 participants.	High rate of exposure to needle stick injuries, and there is a need to promote awareness of PEP protocols.	Not included.
Makhado and Seekane (2020)	To explore the level of knowledge on PEP among nurses.	Africa	Systematic review	EBSCOHost, Sabinet, Google Scholar, ScienceDirect, and PubMed. Seven studies were included.	There is poor knowledge of PEP; there is a need to increase awareness and knowledge.	Only studies done in Africa are included, and it cannot be generalised to other continents. Limited further explanation to some aspects of the review.
Kabotho and Chives (2020)	To approximate the commonness of occupational exposure to HIV, reporting and using PEP, knowledge, attitudes towards HIV PEP and ICP among nurses.	South Africa	Cross-sectional study	Front line nurses. A total of 160 participants.	Half of the participants exposed to needle stick injury reported and went on PEP. Six out of 10 completed their treatment. Only half of the participants knew about PEP.	Not included.
Rasweswe and Peu (2020b)	To investigate how knowledgeable nurses are about HIV PEP in the era of controlled and stable HIV prevalence.	South Africa	Non-experimental, descriptive quantitative survey	Three hundred twenty-one nurses who had been working in three selected hospitals in different wards. A total of 100 participants.	Most nurses are not familiar with most information, such as guidelines to prevent occupational infection. The majority had heard about PEP but were not sure if it was available in their hospitals.	Not included.
Mossburg et al. (2019)	Occupational hazards among healthcare workers in Africa.	Africa	Systematic review	Three databases (PubMed, Embase, and Cumulative Index to Nursing and Allied Health Literature [CINAHL]).	The lifetime prevalence of needlestick injury ranged from 22% to 95%, and 1-year prevalence ranged from 39 to 91%. Studies included a range of descriptive statistics of knowledge, attitudes, practice and access factors related to exposures. Two studies reported that 21% – 32% of respondents linked poor knowledge or training with the prevention of needlestick injuries. Rates of recapping needles ranged from 12% to 57% in four studies. Attitudes were generally positive towards occupational safety procedures whilst access was poor.	It is possible that the data presented here for PEP under-represent published data as PEP was not the review’s primary focus. We did not include a formal method for evaluating the quality of the studies we included, rather because there were so few studies on the topic that included all available published data. Many studies grouped healthcare workers from disparate professions with varying opportunities for exposure, which could affect needlestick injury or mucocutaneous exposure rates.
Mutisya (2021)	Predictors of HIV infection risk among Health-Care Workers in Sub-Saharan Africa.	Sub-Saharan Africa	Literature review		In SSA, HCWs operate in an environment with high cases of HIV infections and are prone to fall victim to such infections themselves.	
Mabina et al. (2018)	To determine and to describe TB/HIV exposure among nursing students in the clinical practice environment.	South Africa	Cross-sectional study	Purposive sampling was used to sample 151 students.	A high percentage of nursing students reported that they have experienced a situation where they were afraid that they might have been infected with both TB and HIV. Awareness about post-exposure prophylaxis (PEP) was marked among the majority of nursing students. The uptake of PEP differed across junior and senior levels of nursing students. The results revealed that the higher the level of study, the greater the exposure.	The findings cannot be generalised to other NWU campuses as only one campus was used for data collection. Time constraints and exams prevented other nursing students to participate in the study.
Mekgoe et al. (2019)	To explore and describe the experiences of nursing students regarding clinical support in the management of TB and HIV patients in the PHC setting.	South Africa	Phenomenological study	12 nursing students (all African, males and females).	Factors inhibiting clinical support in TB and HIV Management; Incompetence of nursing students; Outcomes of clinical support in TB and HIV Management; Promotion of clinical support.	

HIV, human immunodeficiency virus; PEP, post-exposure prophylaxis; PLWH, People Living with HIV.

## Thematic analysis of selected studies

Thematic analysis was adopted as the method of analysis, using Caulfield’s method (Caulfield 2019). Reading the abstracts and full articles helped the researchers familiarise themselves with the data. Following this, the researchers highlighted common words, expressions and findings in the data during coding. From these, the researchers developed themes, as illustrated in Table 3, leading to the results and discussion. The analysis yielded the following themes:

- Theme 1: Knowledge of PEP (*n* = 9) (Aigbodion, Motara & Laher 2019; Bareki & Tenego 2018; Mabina et al. 2018; Makhado & Davhana-Maselesele 2016; Makhado & Seekane 2020; McDowall & Laher 2019; Mutisya 2021; Ncube, Meintjes & Chola 2014; Rasweswe & Peu 2020).
- Theme 2: Attitudes towards PEP (*n* = 4) (Bareki & Tenego 2018; McDowall & Laher 2019; Mekgoe et al. 2019; Ncube et al. 2014).
- Theme 3: Adherence towards and practice of PEP (*n* = 10) (Aigbodion et al. 2019; Bareki & Tenego 2018; Kabotho & Chives 2020; Mabina et al. 2018; Makhado & Davhana-Maselesele 2016; Makhado & Seekane 2020; McDowall & Laher 2019; Mossburg et al. 2019; Ncube et al. 2014; Rasweswe & Peu 2020, 2021).

**TABLE 3:** Emerged themes and sub-themes.

Theme	Sub-themes
Knowledge of PEP	-
Attitudes towards PEP	Positive attitude Stigmatisation
Adherence towards and practices related to PEP	Adherence to PEP

PEP, Post-exposure prophylaxis.

### Ethical considerations

This article followed all ethical standards for research without direct contact with human or animal subjects.

## Results

From the data, two major themes emerged: knowledge of and attitude towards PEP and adherence to PEP (see Table 3). Six studies included here show that most participants in the studies have been previously exposed, some even more than twice. For this reason, it is essential to have adequate knowledge and adherence to PEP. The study found that HCPs and students in training have somewhat sufficient knowledge of PEP; however, adherence to PEP-related protocols is low.

### Theme 1: Knowledge of post-exposure prophylaxis

The included studies commonly found that having adequate knowledge of PEP was characterised by having heard about PEP and having information concerning PEP. On the opposite side, inadequate understanding of PEP would entail never having heard about PEP and having little knowledge and more misconceptions than truths relating to PEP. Furthermore, it was found that among the nine studies on knowledge and awareness of PEP, many of the studies (*n =* 6) reported that most of the participants had adequate knowledge of PEP, with over 60% of their participants having heard of PEP (either formally or informally). On the other hand, some studies (*n =* 4) highlighted that there is some level of knowledge on PEP. Some studies highlighted that though participants may have known about PEP, they have misconceptions regarding its purpose, administration and intake duration (Bareki & Tenego 2018; Makhado & Davhana-Maselesele 2016; Makhado & Seekane 2020; Mabina et al. 2018; Rasweswe & Peu 2020a, 2020b).

In addition to this, some studies expressed a need for education and knowledge sharing regarding PEP and PEP practices and protocols (Bareki & Tenego 2018; Kabotho & Chives 2020; Mabina et al. 2018; Makhado & Davhana-Maselesele 2016; Makhado & Seekane 2020; Rasweswe & Peu 2020a, 2020b). Four of the included studies showed the lack of and need for education on PEP protocol for HCPs (Mabina et al. 2018; Makhado & Davhana-Maselesele 2016; Makhado & Seekane 2020; Rasweswe & Peu 2020a). There is a need for educational methods such as information pamphlets, posters and formal education. The need for education suggests that there is no intentionality in knowledge sharing. Most of the elementary information individuals have on PEP was attained through word of mouth or HCP leaders at their place of practice.

### Theme 2: Attitudes towards post-exposure prophylaxis

Reports on attitudes towards PEP correlate with the suggested satisfactory level of knowledge. Of the included studies that reported on attitudes on PEP (*n =* 4), they reported that people have positive attitudes toward PEP. Most HCPs understood its importance and appreciated reducing HIV contraction during practice to support this conclusion. However, it is unclear if HCPs have a positive attitude because of having a good understanding of PEP or knowing PEP to relieve the possible threat of infection. In this regard, the accuracy of the positive attitude towards PEP may be questionable because of HCPs’ practices toward PEP.

### Theme 3: Adherence towards and practice of post-exposure prophylaxis

As these studies presented themselves, there is little information on procedures that should follow the blood and bodily fluid exposure hence the expressed need for education. Harmful practices towards PEP were mostly characterised by the urgency of taking treatment post-exposure and not knowing where to report exposure. Moreover, being misinformed about the availability of PEP treatment in the facility, ignorance of exposure, and starting treatment late contributed to harmful practices. The common thread in these cases is the inconsistency of treatment availability, accountability and knowledge-sharing concerning the PEP procedure. These may be the determening factors for good PEP practice. One study reported a shortage of PEP medication. Even when they wanted to, they could not get access to treatment. With this said, in most studies, practices towards PEP were characterised by adherence.

In the included studies, four out of nine did not reflect good adherence practice because of insufficient knowledge concerning PEP. However, about six of the included studies showed that HCPs often started PEP treatment post-exposure, with an average percentage completing the course of treatment (55.6%). Some reasons for not adhering to or completing treatment included experiencing side effects, HIV stigma, fear of reporting and HCPs not thinking they needed PEP. In some instances, HCPs stopped PEP treatment because they tested negative, which excused the need to continue treatment. These may result in a lack of overall adherence and momentousness regarding PEP treatment.

## Discussion

Results show that HCPs have adequate knowledge of PEP in Southern Africa, meaning they know what PEP is and measures to take in case of exposure (Aigbodion et al. 2019; Bareki & Tenego 2018; Kabotho & Chives 2020; Makhado & Davhana-Maselesele 2016; Makhado & Seekane 2020; McDowall & Laher 2019; Mossburg et al. 2019; Mutisya 2021; Ncube et al. 2014; Rasweswe & Peu 2020a, 2020b). Although this may be true, having heard about it, PEP may not be an accurate measure of knowledge as it does not include knowledge concerning PEP measures and treatment. In addition to this, among HCPs, stigma and misconception persist. What is more, factors such as inconsistent and unintentional knowledge sharing, lack of emphasis on the importance of PEP and lack of designated medical safety accountability structures may contribute to insufficient knowledge. Any inadequate knowledge on PEP held by medical practitioners is alarming because of the nature of work and recurrent exposure they experience.

It may be assumed that most HCPs have a positive attitude because of the high percentage who have adequate knowledge. However, misconceptions concerning practice persist, making it unclear what the source of a positive attitude is. On the other hand, as alluded by one study, a negative attitude may result if individuals are not necessarily known but are simply aware and do not have the correct information on PEP.

Moreover, general awareness and knowledge within medical facilities are pivotal. Some HCPs may not know the correct protocols to avoid exposure or actions after exposure and treatment to take (Prathapasinghe & Dharmarathne 2018). This study suggests that although HIV stigma, lack of available treatment and fear of exposure may influence adherence, these factors may also influence seeking information regarding post-exposure protocols. As shown in a study by Chalya et al. (2015) in Tanzania, HCPs do not report occupational accidents in fear of the side effects.

As most studies suggested, there is a need for formal education for students as they are more at risk of occupational exposure (Aigbodion et al. 2019; Hada et al. 2018; Matos et al. 2021; Triassi & Pennino 2018). The lack of experience during practicals and the beginning of their careers may be why they are at high risk for exposure. This education will clarify practices, reduce anxiety, increase positive attitudes and decrease misconceptions and stigma associated with HIV among HCPs. This case would assist in knowledge sharing and adherence if information about PEP was already publicised. Although the more significant majority completed the course of the PEP treatment, a high number of HCPs does not finish treatment because of several reasons that are somewhat justified (Aigbodion et al. 2019; Bareki & Tenego 2018; Makhado & Seekane 2020; Ncube et al. 2014; Sultan et al. 2014; Chimoyi et al. 2022). The ill knowledge of PEP procedures shows the importance of follow-ups. It may result in more adherence and decreased side effects of exposure because of provided support, such as psychological support (Vedhanayagam, Sengodan & Rajagopalan 2016; Beekmann & Henderson 2014). A study conducted by Chalya et al. (2015) in Tanzania also discovered that HCPs do not report occupational accidents because of fear of the side effects, as 50% showed that participants knew when and where to report, which medication to take and which protocols to follow in case of exposure. Although knowledge of relevant PEP processes may align with the levels of adherence, most HCPs do not complete the recommended 28-day course of therapy.

### Limitations

This study only included HCPs and students in the Southern African region training. Many of the studies included were quantitative, and perhaps more data would have emerged should there have been qualitative studies within the inclusion criteria. The findings of this study may not be generalised as the included studies may not accurately represent health HCPs (in practice and training) in Southern Africa.

## Conclusion

This review aimed to assess PEP’s knowledge, adherence and practices in Southern Africa. It was found that there is a somewhat reasonable level of PEP, especially regarding whether HCPs have ever heard about PEP. On the other hand, although reports showed adequate knowledge of what PEP is, there is insufficient knowledge regarding PEP processes that may play a role in adherence and measures employed post-occupational exposure.

### Recommendations

It is recommended that the HIV programme managers and supervisors employ follow-ups and accountability measures to increase adherence and completion of PEP. Furthermore, the formal education (pre-service) and HIV training should include PEP protocols and adherence to PEP emphasis. It is also recommended that researchers should consider PEP with fewer side effects than those available to aid adherence and completion. Further research can be done focusing on the relationship between adherence and level of knowledge on PEP. Health institutions should always have PEP available and accessible for all HCPs.
